# The impact of SARS-Cov-2 infection on the periocular injection pain and hypersensitive reaction to botulinum toxin type A: results from clinical questionnaires

**DOI:** 10.3389/fcimb.2023.1240303

**Published:** 2023-09-05

**Authors:** Xiangyu Liu, Yanli Tian, Chanyuan Jiang, Miao Dong, Ming Li, Hefeng Sun, Xuefeng Han, Facheng Li

**Affiliations:** ^1^ Body Sculpture and Fat Transplantation Center, Plastic Surgery Hospital, Chinese Academy of Medical Science and Peking Union Medical College, Beijing, China; ^2^ ADD+ Medical Esthetic Clinic, Beijing, China

**Keywords:** COVID-19, botulinum toxin, periocular wrinkles, hyperalgesia, allergic reaction

## Abstract

**Background:**

The COVID-19 pandemic has brought about significant changes in the medical field, yet the use of botulinum toxin type A has remained uninterrupted. Plastic surgeons must carefully consider the timing of administering botulinum toxin type A to patients who have recovered from COVID-19.

**Methods:**

A questionnaire survey was conducted among patients who had contracted and recovered from SARS-CoV-2 within a month. The survey aimed to investigate various indicators in patients who had received botulinum toxin A injections at the same site before and after their infection, including pain scores and allergic reactions and the occurrence of complications.

**Results:**

The pain scores of patients who contracted SARS-CoV-2 infection between 14-21 days post-infection exhibited significant variation from previous injections. However, patients who contracted the infection between 22-28 days post-infection did not exhibit significant variation from previous injections. Furthermore, the incidence of allergic reactions and complications following botulinum toxin injection within one month after contracting the infection did not significantly differ from that observed prior to infection.

**Conclusion:**

Administering botulinum toxin type A three weeks after COVID-19 recovery is a justifiable and comparatively secure approach.

## Introduction

The Coronavirus Disease 2019 (COVID-19) pandemic has triggered an unprecedented transformation across all domains of medicine, significantly impacting patient management, and necessitating the modification of our clinical and surgical practices. Therefore, it is imperative for plastic surgeons to carefully contemplate the scheduling of elective cosmetic procedures for patients convalescing from COVID-19.

Since its inaugural authorization in 1989, Botulinum toxin type A has emerged as one of the most extensively employed injectables for both cosmetic and therapeutic applications, witnessing an escalating demand globally. Despite the COVID-19 pandemic, the utilization of Botulinum toxin type A remained undeterred. Astonishingly, in the statistics of patients receiving minimally invasive cosmetic procedures in the United States from 2021 to 2022, botulinum toxin type A treatment ranks first (American Society of Plastic Surgeons, ).

Current literature indicates that COVID-19 frequently manifests with symptoms such as myalgia, pain, and pervasive hyperalgesia (El-Tallawy et al., 2020). Severe Acute Respiratory Syndrome Coronavirus 2 (SARS-CoV-2), the causative agent of COVID-19, may instigate an augmented secretion of pro-inflammatory cytokines, attracting other pro-inflammatory cells such as granulocytes and macrophages. This provokes a cascade effect, escalating cytokine secretion and leukocyte recruitment, thereby instigating a systemic inflammatory response. The plasma concentration of several cytokines, including IL-2, IL-6, IL-1β, IL-1RA, IL-7, IL-8, IL-10, IFN-ɣ, MCP-1, MIP-1α, G-CSF, and TNF-α, witness an upsurge (Huang et al., 2020; Anka et al., 2021). Particularly, IL-6, a critical inflammatory cytokine, is implicated in muscle pain, and intraplantar administration of IL-6 to mice induces mechanical hyperalgesia (Xu et al., 1997; Dina et al., 2008; Akdogan, 2021). A case report highlighted a 55-year-old female patient experiencing severe hyperalgesia and pain during a botulinum toxin injection a week following her recovery from COVID-19. The patient rated her pain severity as 10 on a 10-point scale, in stark contrast to a rating of 3 for a similar procedure conducted four months prior (Akdogan, 2021). Furthermore, two sub-acute cases of hypersensitive reactions to Chinese BTA (marketed as Prosigne in Brazil, Lanzhou Institute of Biological Products, China) were documented in China post COVID-19 vaccination (Sabico et al., 2021).

In December 2022, a sudden and large-scale SARS-CoV-2 infection occurred among the populace in China. As the virus exhibited a diminished potency, the majority experienced mild symptoms and recuperated within a fortnight, subsequently seeking botulinum toxin type A injections per their routine. Consequently, the impact of COVID-19 on the pain and hypersensitivity reaction following botulinum toxin injection warrants comprehensive exploration. The objective of our research is to determine whether significant disparities exist in the manifestation of pain and other hypersensitive reactions in patients subjected to periocular injections following a SARS-CoV-2 infection.

## Materials and methods

### Database and study population

A questionnaire-based survey was conducted among patients from December 2022 to February 2023 at the Plastic Surgery Hospital of the Chinese Academy of Medical Sciences and ADD+ Medical Esthetic Clinic in China. Inclusion criteria encompassed: (1) prior receipt of Botulinum toxin A (Botox; Allergan Inc.) injections at least once by the same practitioner at the same institution; (2) documented SARS-CoV-2 infection and recovery within a month, with recovery criteria entailing the absence of fever, dyspnea, fatigue, cough, headache, sore throat, body aches, chills, anosmia, ageusia (Pan et al., 2020; Sabico et al., 2021); and (3) willingness to participate in the survey as a healthy individual. Participants were informed and provided consent to partake in the study. This research was approved off by the Ethics Committee of the Plastic Surgery Hospital of the Chinese Academy of Medical Sciences.

The survey was administered by four resident physicians and two graduate students, all of whom had undergone standard training prior to the survey to ensure comprehension of survey methods, questionnaire distribution techniques, data acquisition protocols, and guidelines for data cleansing and organization. Each physician was assisted by 2-3 investigators during the visit. Potential participants were informed about the survey’s purpose, and the paper-based questionnaire was provided after securing informed consent One investigator clarified the survey items to the participants. Upon completion of the questionnaire, two investigators simultaneously collected the data. During the three-month study period, a total of 150 questionnaires were disseminated, and 146 questionnaires were retrieved, with four patients failing to complete the questionnaire. The survey response rate was 97.3%. The questionnaire comprised two parts:

(1) patient demographics such as name, age, gender, timeline of COVID-19 infection, previous and current botulinum toxin injection sites. The treatment sites investigated included lateral canthal lines, glabellar frown lines, nasal dorsal lines, and forehead wrinkles. Each treated site was considered as a standalone treatment unit for statistical evaluation. Treatment units that had been addressed in both previous and current sessions were included in the analysis.
(2) Patients were asked to score the pain level (0 for painless, 10 for severe pain) for each treatment unit during both past and present injections. Any allergic reaction or complication occurring during the present or previous injection for each treatment unit was also documented. The allergic reaction survey included inquiries about localized swelling, itching, red bumps, and systemic breathing difficulties. The complication survey queried the presence of ptosis, strabismus, diplopia, eyelid paralysis, and brow ptosis.

### Statistical analysis

Continuous variables were compared by Student’s t-test for variables with normal distribution, or the Mann–Whitney U test for variables without normal distribution; categorical variables were compared by Pearson’s Chi-square test or Fisher’s exact test. Two-sided p-values <0.05 were considered statistically significant. Analyses were performed using the software packages SPSS v.19.0 (SPSS, IL, USA), Stata v.12.0 (StataCorp, Texas, USA) and R v.3.2.2 (R Foundation for Statistical Computing, Vienna, Austria).

## Results

The study encompassed a total of 146 subjects, with an average age of 41.0 ± 9.7 years, which include 12 males (12/146) and 134 females (134/146) (**Table 1**). The average duration post COVID-19 infection was 21.3 ± 4.3 days. Two patients exhibited local allergic reactions and no patients developed complications (**Table 2**). One patient developed urticaria-like plaques around the injection site five minutes post lateral canthal line injection, which resolved spontaneously three hours post injection. Interestingly, the patient had exhibited similar symptoms during their prior treatment. Another patient developed mild upper eyelid edema and periocular itching in the right eye eight hours post lateral canthal line injection, which resolved after three days of oral anti-allergy medication (Loratadine Tablets) as prescribed. No previous local allergic reaction was reported for this patient. Despite the allergic reactions, neither patient’s treatment outcomes were affected. No systemic allergic reactions were reported. The analysis showed no significant difference in the incidence of allergic reactions to botulinum toxin injections pre and post SARS-CoV-2 infection. No complications were noted post injection, nor were any previous complications reported in this study. The analysis revealed no significant difference in the incidence of complications between botulinum toxin injections pre and post SARS-CoV-2 infection.

**Table 1:** Summary of demographic characteristics and descriptive results.

Population	146
Age	41 ± 9.7
Gender	
Male	12
Female	134
Total treatment times	399
Days since COVID-19 Infection	21.3 ± 4.3
Complication cases	0
Hypersensitive cases	2

**Table 2:** Information of patients with hypersensitive reaction.

	Age	Gender	Site	History	Symptom	Treatment	*p*
Patient 1	23	F	lateral canthal lines	—	5 minutes after surgery, urticaria-like plaques appear around the injection site	—	>0.05
Patient 2	25	F	lateral canthal lines	—	8 hours after surgery, right upper eyelid edema and periocular itch	Loratadine Tablets	

The analysis divided the time since SARS-CoV-2 infection into two periods: 14-21 days and 22-28 days.

**Figure 1:**
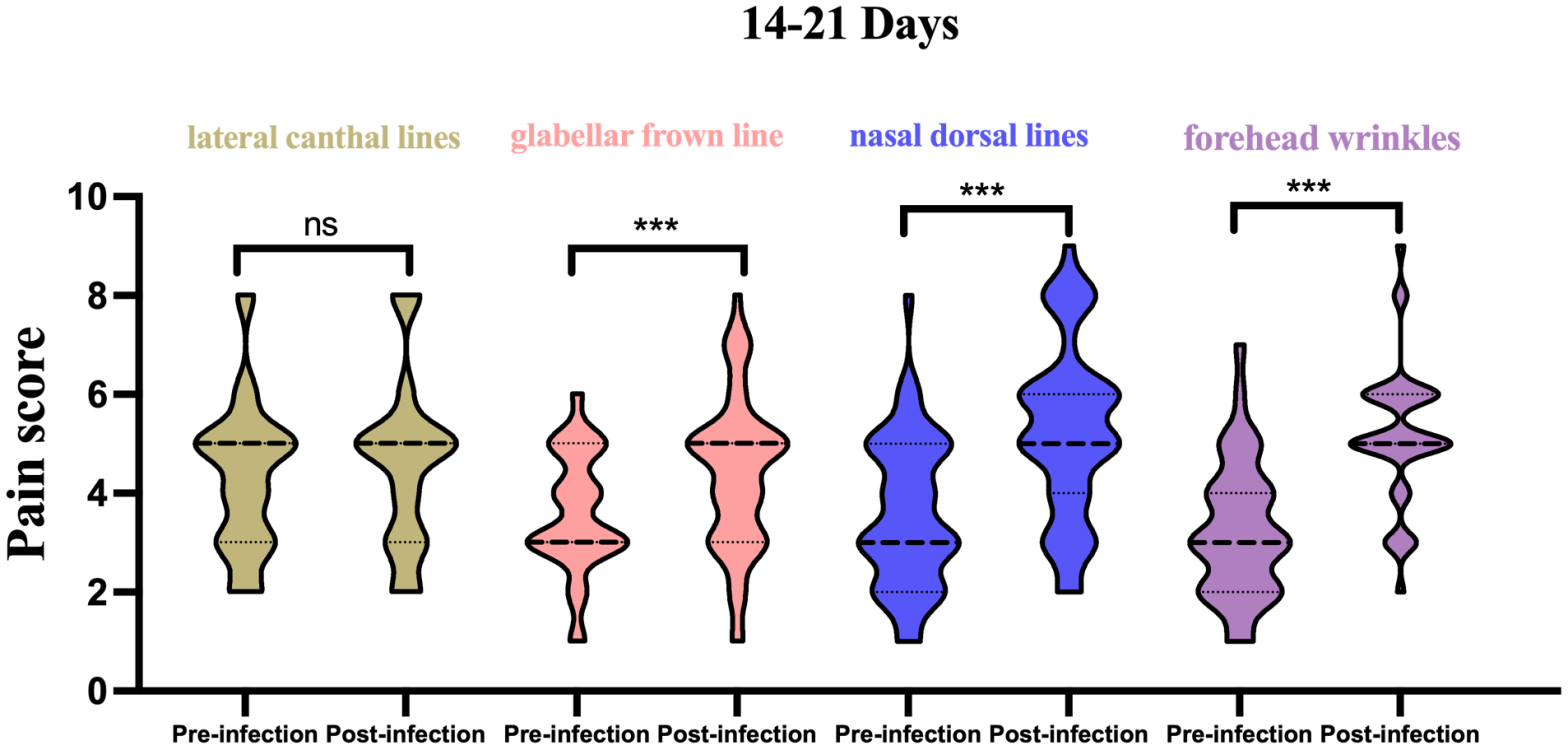
Histogram of pain scores from pre vs. post infection in 14-21 days, stratified by injection site. ***p<0.05; ns, not significant.

**Figure 2:**
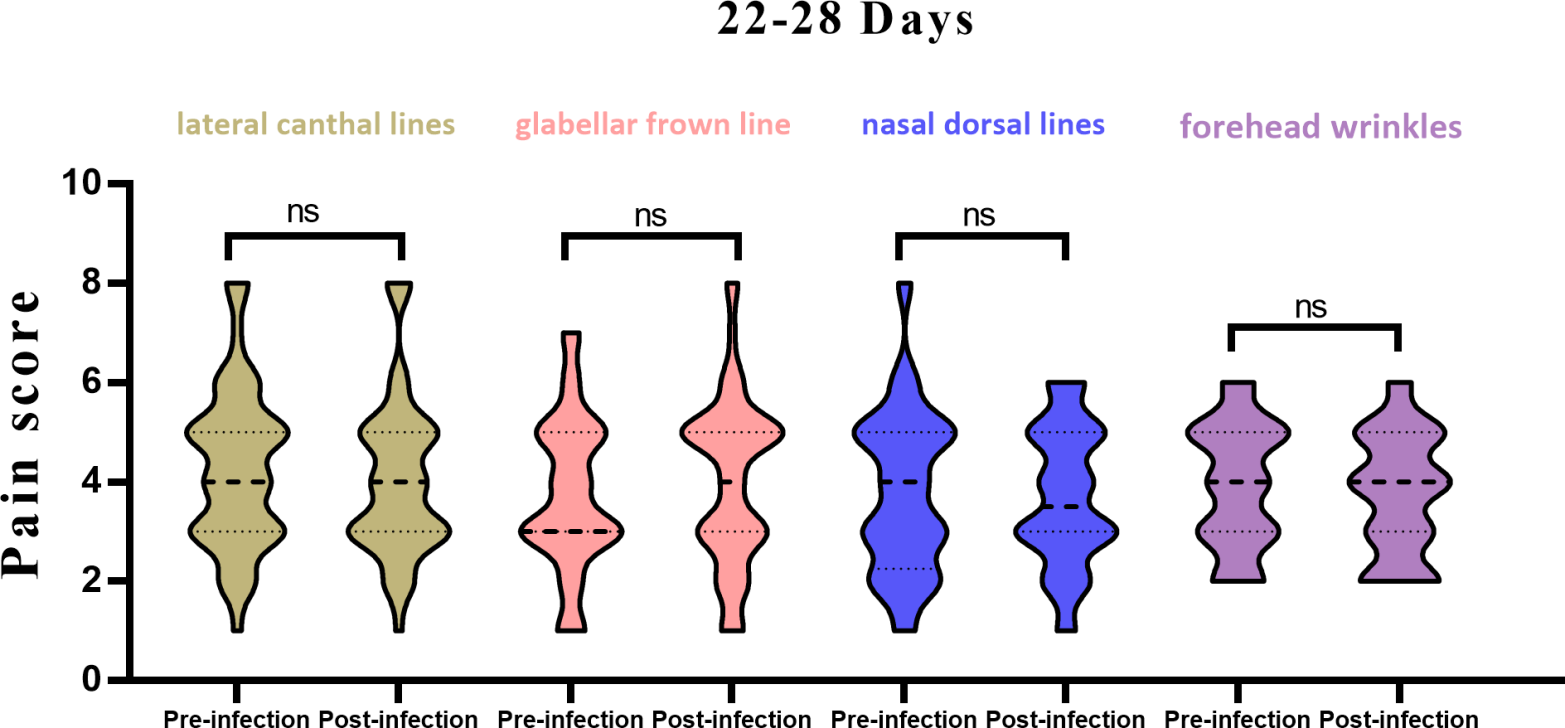
Histogram of pain scores from pre vs. post infection in 22-28 days, stratified by injection site. ns, not significant.

(1) In the 14–21-day window: For the lateral canthal lines unit, the pain score was 3.4 ± 1.5 on average for previous injections, and 3.4 ± 1.7 for the current treatment, indicating no significant difference. For the glabellar frown lines unit, the average previous pain score was 3.6 ± 1.2, and 4.4 ± 1.5 for the current treatment, showing a significant difference. For the forehead wrinkles unit, the average previous pain score was 3.6 ± 1.5, and 5.2 ± 1.8 for the current treatment, displaying a significant difference. For the nasal wrinkle unit, the average previous pain score was 3.2 ± 1.4, and 5.2 ± 1.4 for the current treatment, also revealing a significant difference (**Figure 1**).

(2) In the 22–28-day window: For the lateral canthal lines unit, the average previous pain score was 4.3 ± 1.6, and 4.1 ± 1.6 for the current treatment, showing no significant difference. For the glabellar frown lines unit, the average previous pain score was 3.7 ± 1.6, and 4.0 ± 1.7 for the current treatment, again demonstrating no significant difference. For the forehead wrinkles unit, the average previous pain score was 3.8 ± 1.6, and 3.7 ± 1.4 for the current treatment, indicating no significant difference. For the nasal wrinkle unit, the average previous pain score was 3.9 ± 1.3, and 3.8 ± 1.2 for the current treatment, also indicating no significant difference (**Figure 2**).

**Figure 3:**
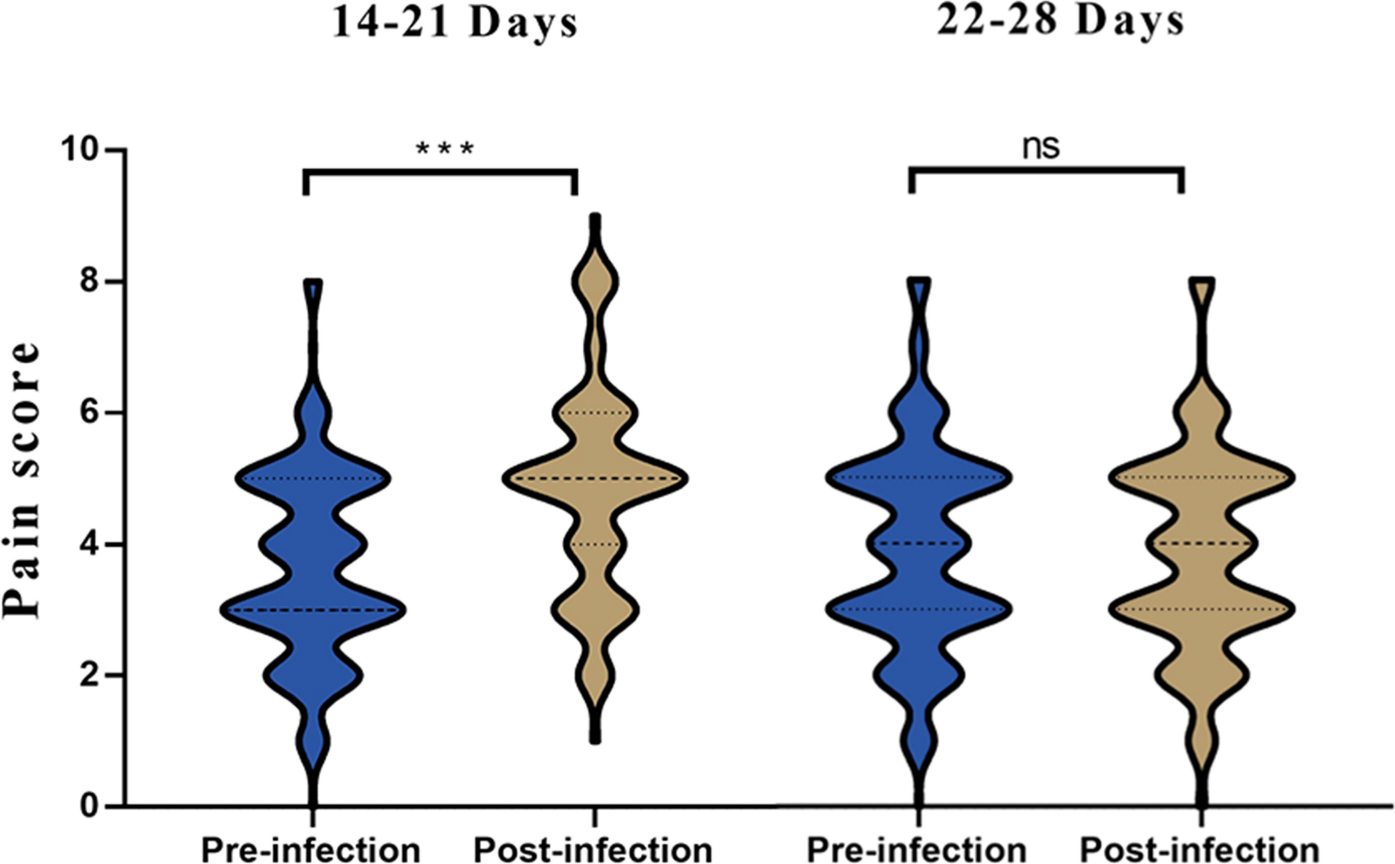
Histogram of pain scores from pre vs. post infection in 14-21 and 22-28 days. ***p<0.05; ns, not significant.

These findings suggest that, while there were significant differences in pain scores for the glabellar frown lines, forehead wrinkles and nasal dorsal lines units between 14-21 days after infection, no significant differences were observed 22-28 days after infection across all treatment units (**Table 3**). Significant differences were observed when comparing previous and current pain scores for all treatment units for patients 14-21 days post SARS-CoV-2 infection (**Figure 3**). However, no significant differences were observed when comparing previous and current pain scores for all treatment units for patients 22-28 days post SARS-CoV-2 infection.

**Table 3:** Summary of pain score results, stratified by time since COVID-19 infection and injection site.

Infection time	Site	Treatment times	Current pain score	Initial pain score	*p*
14-21 days	lateral canthal lines	54	3.4±1.7	3.4±1.5	>0.05
	glabellar frown lines	52	4.4±1.5	3.6±1.2	<0.01
	forehead wrinkles	53	5.2±1.8	3.6±1.5	<0.01
	Nasal dorsal lines	41	5.2±1.4	3.2±1.4	<0.05
22-28 days	lateral canthal lines	54	4.1±1.6	4.3±1.6	>0.05
	glabellar frown lines	54	4.0±1.7	3.7±1.6	>0.05
	forehead wrinkles	58	3.7±1.4	3.8±1.6	>0.05
	Nasal dorsal lines	33	3.8±1.2	3.9±1.3	>0.05

## Discussion

Botulinum toxin, a protein and neurotoxin produced by the bacterium Clostridium botulinum (Mya et al., 2019), inhibits the release of acetylcholine—a key neurotransmitter at the neuromuscular junction—from cholinergic neurons, thus resulting in muscle paralysis. This effect, when used in a cosmetic context, leads to the selective relaxation of muscles and consequently, the reduced visibility of wrinkles, making it a popular choice for cosmetic procedures. The world faced a new pandemic with the emergence of COVID-19, caused by SARS-CoV-2, as declared by the World Health Organization (WHO) on March 11, 2020 (Ghebreyesus, 2020). While research has revealed that the lungs are the primary target of SARS-CoV-2 (Mohseni Afshar et al., 2021; Mohaghegh et al., 2022), the virus also triggers a range of other systemic reactions, including both localized and generalized complications (Miladi et al., 2021). There have been numerous reports of cutaneous reactions in skin areas previously treated with cosmetic materials. Such SARS-related reactions in injection sites have included induration, edema, tenderness, erythema, discoloration, and nodule formation (Rowland-Warmann, 2021; Shome et al., 2021; Munavalli et al., 2022). When considering allergic reactions to Botox in cosmetic settings, a total of six cases have been reported to date (Rosenfield et al., 2014; Careta et al., 2015; Moon et al., 2017; Sudre et al., 2021). Of these, four patients experienced localized symptoms, while two had generalized symptoms. Two patients exhibited allergies to Botox/Vistabel, three had reactions to Prosigne, and one patient, unfortunately, died from an unspecified BTA. Furthermore, two patients reported facial swelling and flu-like symptoms following the injection of a Chinese BTA. They had received the SARS-CoV-2 Vaccine (Vero Cell, an inactivated vaccine, produced by Sinovac Life Sciences Co., LTD, Beijing, China) two weeks prior to the BTA injection. Both patients had previously undergone BTA injections for the treatment of masseter hypertrophy without any adverse events.

Although allergic reactions to Botulinum toxin are documented in literature, they are exceedingly rare (Rosenfield et al., 2014). Allergic responses can vary widely, ranging from non-serious skin rashes to more serious skin rashes, granuloma formation, and even localized or systemic anaphylactic reactions. It remains uncertain whether these anaphylactic reactions are triggered by the neurotoxin itself or its reconstitution solvent (LeWitt and Trosch, 1997; Li et al., 2005; Ahbib et al., 2006). The underlying mechanism involves an immunologic response to the viral spike protein, leading to cutaneous reactions (Munavalli et al., 2021). Despite raising significant concern, most filler reactions following a SARS-CoV-2 infection have been short-lived and self-limiting. For prolonged or severe cases, therapeutic options have included antihistamines, nonsteroidal anti-inflammatory drugs (NSAIDs), corticosteroids, angiotensin-converting enzyme (ACE) inhibitors like Lisinopril, colchicine, 5-fluorouracil, and methotrexate.

In our study, one patient developed urticaria-like plaques around the injection site five minutes post-injection on the lateral canthal lines. The symptoms, however, subsided on their own within three hours without any intervention. Another patient developed right upper eyelid edema and periocular itch eight hours post-injection on the lateral canthal lines, which was relieved within three days using Loratadine tablets. The treatments for both patients were successful in achieving wrinkle reduction, with patients expressing satisfaction with the outcome. Our analysis revealed no significant difference in the incidence of complications between botulinum toxin injections administered before and after a SARS-CoV-2 infection.

Upon exposure to a virus or viral antigen, both innate and adaptive immune cells collaborate to mount an antiviral response (Felsenstein et al., 2020). Upon exposure to a virus or viral antigen, both innate and adaptive immune cells work together to mount an antiviral response (Felsenstein et al., 2020). The most severely affected patients often exhibit a ‘cytokine storm’ (CS) characterized by elevated pro-inflammatory cytokine levels in the serum (Huang et al., 2020; Qin et al., 2020). One key player in CS, Interleukin-6 (IL-6), shows elevated levels in both mild and severe SARS-CoV-2 infected patients. However, a significant increase in this cytokine is noted in patients with severe disease progression compared to those with mild or non-severe SARS-CoV-2 infections (Crayne et al., 2019; Qin et al., 2020). Studies by Diao et al. and Wan et al. demonstrated a correlation between lymphopenia and IL-6, IL-10, and TNF-α, while restored bulk T cell frequencies, paired with generally lower pro-inflammatory cytokine levels, were observed in convalescent patients (Diao et al., 2020; Wan et al., 2020).

In our research, we observed significant differences in the analysis of pre- and post-infection pain scores across all treatment units for patients 14-21 days post-SARS-CoV-2 infection. However, no such differences were seen in patients 22-28 days post-infection. For the 14-21 day group, notable differences between the two pain scores were identified in the treatment units for glabellar frown lines, forehead wrinkles, and nasal wrinkles. A case report highlights a 55-year-old female patient who experienced severe hyperalgesia and pain during botulinum toxin injection one-week post-recovery from a COVID-19 infection. Her pain severity, rated on a 10-point scale, was 10 during this session compared to a score of 3 during her last session, performed four months prior (Akdogan, 2021). The average recovery time from COVID-19 is 2–3 weeks, depending on symptom severity (Daher et al., 2020; Burn et al., 2021). Post-infection symptoms include shortness of breath, muscle pain, joint pain, headache, cough, chest pain, altered smell, altered taste, and diarrhea. Cognitive impairment, memory loss, anxiety, and sleep disorders are other common symptoms (Aiyegbusi et al., 2021). We interpret the injection time range in this study to coincide with the average recovery time from COVID-19 (24 ± 4.1 days), given that patients might avoid seeking cosmetic treatment due to uncomfortable symptoms. This observation also suggests that the elevated levels of IL-6, a significant contributor to CS, could have led to the severe hyperalgesia and pain experienced by the 55-year-old female patient.

With the rising number of SARS-CoV-2 infections, we are witnessing an increasing number of reported adverse events associated with botulinum toxin A (BTA) treatments. This correlation underscores the importance of understanding the potential complications and long-term safety of these treatments (Wang et al., 2021). Although this study cannot pinpoint an exact and safe treatment window, we propose that BTA injections for cosmetic purposes in SARS-CoV-2 infected individuals should ideally be carried out no earlier than three weeks post-infection. Despite the small sample size of this study and the occurrence of cases with mild symptoms and quick recoveries, these factors are insufficient to deter BTA-related cosmetic injections in the context of SARS-CoV-2 infection. We recommend the selection of a safer and more reasonable injection timeframe, prioritizing patient safety above all. Preoperative evaluation and timely postoperative follow-up should be routine practices.

Limitations of this study include: a sudden surge of SARS-CoV-2 infections in December, which limited the number of individuals who met the study criteria. Most patients in the study resided in Beijing, and the majority of the coronavirus subtypes were BF7. The botulinum toxin brand used in this study was BTA (Botox; Allergan, Inc.), leaving no opportunity to study other brands. While the injections were performed by the same physician who had administered BTA several times before, individual injection methods varied due to differences in wrinkles presented by each patient, introducing a variable that could not be completely controlled (Li et al., 2022). Additionally, as the patients were only undergoing outpatient injection treatments, no blood analysis of inflammation and allergy factors was conducted to better analyze the correlation between pain and allergies. Inherent limitations of survey research apply here as well, particularly whether respondents answered survey questions honestly. The mention of SARS-CoV-2 infection in the questionnaire and recalling pre-infection scores may have psychologically influenced the pain scores reported by subjects. We sought to mitigate this by avoiding mention of COVID-19 when asking about pain scores and inquiring about the pain score of the pre-infection injection prior to this injection, to minimize this study’s uncontrollable impact. In this study, the duration of the participants’ vaccination was not collected. Future prospective studies will be designed to further optimize and refine the conclusions of this study.

## Conclusion

This study found a significant difference in pain scores between patients 14-21 days post-SARS-CoV-2 infection and their prior injection experiences. However, for patients 22-28 days post-infection, the pain scores were not significantly different from prior instances. The incidence of allergic reactions and complications within one-month post-infection did not significantly differ from pre-infection rates following botulinum toxin injections. We suggest that administering botulinum toxin type A injections three weeks post-recovery from COVID-19 is relatively safe and reasonable. This study provides an essential reference for plastic surgeons when determining the optimal timing for botulinum toxin type A injections in their patients.

## Data availability statement

The raw data supporting the conclusions of this article will be made available by the authors, without undue reservation.

## Ethics statement

The studies involving humans were approved by the Ethics Committee of the Plastic Surgery Hospital of the Chinese Academy of Medical Sciences. The studies were conducted in accordance with the local legislation and institutional requirements. The participants provided their written informed consent to participate in this study. Written informed consent was obtained from the individual(s) for the publication of any potentially identifiable images or data included in this article.

## Author contributions

XL, YT: subjects enrollment, statistical analysis, and draft manuscript. CJ, HS: conceptualization, methodology, statistical analysis, writing, and editing. MD, ML: statistical analysis. All authors contributed to the article and approved the submitted version.
